# Ranking factors affecting emissions of GHG from incubated agricultural soils

**DOI:** 10.1111/ejss.12143

**Published:** 2014-06-18

**Authors:** S García-Marco, S R Ravella, D Chadwick, A Vallejo, A S Gregory, L M Cárdenas

**Affiliations:** a Departamento de Química y Análisis Agrícola, Escuela Universitaria de Ingeniería Técnica Agrícola, Universidad Politécnica de MadridCiudad Universitaria, 28040, Madrid, Spain; b Rothamsted ResearchNorth Wyke, Okehampton, Devon, EX20 2SB, UK; c Departamento de Química y Análisis Agrícola, Escuela Técnica Superior de Ingenieros Agrónomos, Universidad Politécnica de MadridCiudad Universitaria, 28040, Madrid, Spain; d Rothamsted ResearchWest Common, Harpenden, Hertfordshire, AL5 2JQ, UK

## Abstract

Agriculture significantly contributes to global greenhouse gas (GHG) emissions and there is a need to develop effective mitigation strategies. The efficacy of methods to reduce GHG fluxes from agricultural soils can be affected by a range of interacting management and environmental factors. Uniquely, we used the Taguchi experimental design methodology to rank the relative importance of six factors known to affect the emission of GHG from soil: nitrate (NO_3_^−^) addition, carbon quality (labile and non-labile C), soil temperature, water-filled pore space (WFPS) and extent of soil compaction. Grassland soil was incubated in jars where selected factors, considered at two or three amounts within the experimental range, were combined in an orthogonal array to determine the importance and interactions between factors with a L_16_ design, comprising 16 experimental units. Within this L_16_ design, 216 combinations of the full factorial experimental design were represented. Headspace nitrous oxide (N_2_O), methane (CH_4_) and carbon dioxide (CO_2_) concentrations were measured and used to calculate fluxes. Results found for the relative influence of factors (WFPS and NO_3_^−^ addition were the main factors affecting N_2_O fluxes, whilst glucose, NO_3_^−^ and soil temperature were the main factors affecting CO_2_ and CH_4_ fluxes) were consistent with those already well documented. Interactions between factors were also studied and results showed that factors with little individual influence became more influential in combination. The proposed methodology offers new possibilities for GHG researchers to study interactions between influential factors and address the optimized sets of conditions to reduce GHG emissions in agro-ecosystems, while reducing the number of experimental units required compared with conventional experimental procedures that adjust one variable at a time.

## Introduction

Agriculture directly contributes between 5.1 and 6.1 Pg CO_2_-equivalents (eq) (10–12%) to global greenhouse gas (GHG) emissions (Smith *et al.*, 2007). These emissions are mainly in the form of methane (CH_4_) (up to 3.3 Pg CO_2_-eq year^−1^), nitrous oxide (N_2_O) (up to 2.8 Pg CO_2_-eq year^−1^) and carbon dioxide (CO_2_) (few comparable estimates exist) (Smith *et al.*, 2007). However, agriculture has the potential to mitigate these emissions by using existing agricultural technology, with considerable potential in adapting cropland management practices (Smith *et al.*, 2007).

Understanding the processes responsible for GHG emissions from agricultural soils, the major controlling factors and which combination of factors minimize emissions is critical in the development of effective mitigation. It is possible that management and fertilizer practices conducted to reduce one GHG could favour conditions for production and emission of another. Nitrous oxide can be produced by nitrification, incomplete denitrification, nitrifier denitrification and/or fungal denitrification, as well as during reduction of nitrate (NO_3_^−^) to ammonium (NH_4_^+^) (Kool *et al.*, 2010). Methane is produced by methanogenic microorganisms in anaerobic micro-sites (Chan & Parkin, 2001) and consumed by soil methanotrophs, especially in aerobic micro-sites (McLain & Martens, 2006). Carbon dioxide, the other main GHG generated by soils, is produced by autotrophic respiration (roots and associated mycorrhizae) and heterotrophic respiration (soil macro- and micro-fauna) (Hanson *et al.*, 2000). The link between the N and C cycles in these pathways and the influence of different environmental and soil variables in each of these processes introduce a large number of factors, which affect fluxes from agro-ecosystems (Robertson & Groffman, 2007). This complexity is especially important for N_2_O emissions because of its large global warming potential. Thus, Snyder *et al.* (2009) listed the most important factors for N_2_O emissions as (i) soil physical and chemical properties, including organic C content, mineral N content, pH, texture, drainage, temperature, moisture content, O_2_ status, porosity, microbial abundance and activity, and (ii) management-related factors, including N application rate per fertilizer type, fertilizer application technique, application timing, tillage system, irrigation, incorporation of crop residues and type of crop. Many of these factors also affect CH_4_ and CO_2_ emissions.

The individual effects of the major controlling factors on GHG emission processes are well known from laboratory-based and field studies. However, a lack of knowledge exists on how the interactions between those factors affect emissions and how to best optimize conditions to mitigate fluxes.

Laboratory experimentation offers the possibility of a better evaluation of treatment effects because it is easier to control environmental and soil variables (Schaufler *et al.*, 2010). However, conventional experimental approaches that involve altering one factor at a time while keeping all other factors constant are difficult because of the large number of experimental units this statistical approach generates. They are also time consuming and are unable to provide the wider range of combinations of interacting factors of interest.

The experimental design (design of experiments, DOE) used in the Taguchi methodology is a different approach. It estimates the effects of the main factors and the mutual interaction between the chosen factors, and determines the optimum experimental conditions using only a portion of the total possible combinations of a large number of factors (Taguchi, 1987; Rao *et al.,* 2008). While traditional experimental design focuses on the average process performance characteristics, this approach concentrates on the effect of variation on the process characteristics (Phadke, 1989; Ross, 1996) and makes the product and or process performance insensitive to variation by proper design of factors.

In this approach, factors are arranged in an orthogonal array (OA) to reduce experimental errors and to enhance the efficiency and reproducibility of laboratory experiments (Rao *et al.*, 2008). The OA properties are such that between each pair of columns each combination of levels appears an equal number of times. There are several design arrays denoted by a subscript such as L_4_, L_8_, L_9_, L_12_, L_16_, L_18_, L_27_ and L_64_ and these arrays indicate the main information on the extent of the experiment. For example, L_16_ has 16 trials, units, experimental conditions or combinations of these. Because of the orthogonal layout, the effects of the other factors can be balanced to give a relative value representing the effects of a level to be compared with the other levels of a given factor (Taguchi, 1987). In OA experiments, the number of test runs or experimental conditions is minimized while keeping the pair-wise balancing property (Byrne & Taguchi, 1987).

The Taguchi method has been mainly and successfully applied to improve the quality of manufactured products. To date it has been used to limited extent only to determine the optimum process parameters in different areas, including microbial fermentations, molecular biology, food processing, waste water treatment and bioremediation (Rao *et al.*, 2008), and also to study soil erosion (Sadeghi *et al.*, 2012).

To the best of our knowledge, no application of this methodology to the study of GHG emissions has been reported until the present time. The Taguchi approach could be useful to determine optimum conditions to minimize the fluxes, relating these factors and their corresponding levels, and reducing the number of experiments required.

The specific objective of this investigation was to apply, for the first time, the Taguchi orthogonal experimental design to rank the relative importance of different factors on GHG emission processes from agricultural soils, and determine which set of conditions are required to minimize GHG fluxes. A soil incubation experiment (using grassland soil) with five of the most important controlling factors (addition of nitrate, carbon (C) quality, soil temperature, soil moisture as percentage of water-filled pore space (WFPS) and rate of soil compaction) with an OA layout of L_16_ was performed using two or three levels of each factor. Our aim was to identify the relative importance of each factor in the emission of GHG from agro-ecosystems in order to establish mitigation strategies based on agricultural management practices.

## Materials and methods

### Experimental design

This incubation study was designed by following the DOE methodology and adopting the Taguchi approach. This approach comprises the different phases (with various steps) of planning, conducting, analysis and implementation.

In the Taguchi method, performance is measured by the deviation of a characteristic from its target value and a loss function [*L*(*y*)] is developed for the deviation (Ross, 1996), as represented by:


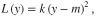
(1)

where *k* denotes the proportionality constant, *m* represents the target value and *y* is the experimental value obtained for each trial. In the case of looking for the quality characteristic of ‘smaller is better’, *m* is equal to 0 and the loss function can be written as:


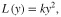
(2)

and the expected loss function can be represented by:


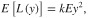
(3)

where *E*(*y^2^*) can be estimated from a sample of *n* as:


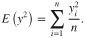
(4)

### Phase I: Planning (selection of factors, levels and OA)

The first step in phase I of the experimental methodology was to identify the important factors (operating parameters) to be tested whose variations are known to have a critical effect on the overall performance of GHG emissions from soils. Six factors, known to influence GHG fluxes significantly (Robertson & Groffman, 2007; Snyder *et al.*, 2009), were considered: concentration of NO_3_^−^, C:N ratio adding glucose as labile C, C:N ratio adding cellulose as non-labile C, soil temperature, soil moisture (as percentage of WFPS) and level of soil compaction (Table 1 and Table S1). Nitrate, glucose and cellulose each had three rates or ‘levels’ assigned (3^3^ in the OA layout), and the soil temperature, percentage of WFPS and soil compaction each had two levels assigned (2^3^ in the OA layout) (Table 1 and Table S1). These levels were chosen according to the expert opinion of the authors, results from previous experiments and published literature on known individual controls (Cárdenas *et al.*, 2003; Robertson & Groffman, 2007; Snyder *et al.*, 2009).

**Table 1:** Selected factors and their levels (rates) assigned to different soil columns used in the Taguchi experiment

	Level 1	Level 2	Level 3
Factors	Low	Medium/high	High
Nitrate (KNO_3_) / kg N ha^−1^	25	50	75
Glucose g C g^−1^ N	C : N = 0	C : N = 5	C : N = 10
Cellulose g C g^−1^ N	C : N = 0	C : N = 5	C : N = 10
Temperature / °C	15	25	—
WFPS / %	< 80	> 80	—
Soil compaction / kPa	50	200	—

The number of factors and levels determined the selected experimental design, which was a L_16_ OA, therefore comprising 16 experimental units. In the next step, an experimental matrix was designed (L_16_-16 experimental units, with a layout of 2^3^ × 3^3^ (Level^Factor^ × Level^Factor^)). The total degrees of freedom for the studied experimental design was 15 (the number of experimental units minus one). Table 2 shows the combination of levels assigned to each factor for the 16 experimental units used in the study. The experimental conditions were obtained by combining Table 1 and the L_16_ OA shown in Table 2.

**Table 2:** Orthogonal array L_16_ (2^3^ × 3^3^) of DOE (Taguchi methodology) and average of cumulative fluxes (after 16 days) as N_2_O, CH_4_, CO_2_ and total GHG as CO_2_ equivalents, (mean ± SEM, *n* = 2)

							Cumulative fluxes			
							
	Levels of factors						N_2_O	CH_4_	CO_2_	CO_2_ equivalents
			
Experimental unit	Nitrate	Glucose	Cellulose	Temperature	WFPS	Soil compaction	/ µg kg^−1^ dry soil		/ mg kg^−1^ dry soil	
1	1	1	1	1	1	1	272 ± 288	−131 ± 26	274 ± 26	356 ± 116
2	1	2	2	1	1	2	129 ± 136	84 ± 95	394 ± 17	435 ± 23
3	1	3	3	2	2	1	9410 ± 887	373 ± 426	1000 ± 327	3925 ± 610
4	1	1	1	2	2	2	9154 ± 566	−98 ± 106	356 ± 14	3191 ± 159
5	2	1	1	1	2	1	5864 ± 161	171 ± 169	328 ± 45	72150 ± 1
6	2	2	3	1	2	2	22 075 ± 1228	53 ± 202	335 ± 115	7180 ± 262
7	2	3	2	2	1	1	378 ± 544	347 ± 1	738 ± 85	862 ± 254
8	2	1	1	2	1	2	116 ± 14	173 ± 59	547 ± 10	586 ± 13
9	3	1	2	2	2	2	73 949 ± 8316	181 ± 61	509 ± 18	23 437 ± 2595
10	3	2	1	2	2	1	35 902 ± 5469	181 ± 63	935 ± 88	12 069 ± 1605
11	3	3	1	1	1	2	337 ± 94	180 ± 42	845 ± 78	953 ± 50
12	3	1	3	1	1	1	294 ± 170	170 ± 81	396 ± 42	490 ± 9
13	1	1	3	2	1	2	234 ± 80	40 ± 130	512 ± 84	585 ± 57
14	1	2	1	2	1	1	275 ± 246	127 ± 5	525 ± 11	613 ± 65
15	1	3	1	1	2	2	15 684 ± 7002	184 ± 37	300 ± 4	5166 ± 2165
16	1	1	2	1	2	1	1879 ± 780	−91 ± 4	259 ± 100	840 ± 141
*Grand average* (mean ± SEM, *n* = 16)							10 997 ± 4902	142 ± 28	516 ± 60	3927 ± 1528

### Phase II: Conducting the designed experiments (soil incubations and GHG emission measurement)

Soil was sampled from the top layer (0–10 cm) of a 1-ha field plot of permanent grassland on the Rowden experimental platform at Rothamsted Research, North Wyke in Devon, UK (50:46:10°N and 3:54:05°W). The soil is a clayey pelostagnogley of the Hallsworth series in the Soil Survey of England and Wales system (Clayden & Hollis, 1984) (a Dystric Gleysol in the FAO, 2006 classification) (Table S2). In order to obtain two ‘field’ replicates, two batches of soil were prepared and kept separate; soil samples from five (field replicate 1) and four (field replicate 2) different points of a ‘W’-shaped transect were taken and pooled in separate containers. Both field replicates were immediately transported to the laboratory, sieved (< 6 mm) whilst still in a field-moist condition and then stored at 2–4°C until required.

Approximately 270 g of fresh soil was packed into a cylindrical plastic core (6.3 cm diameter, 10 cm height) and compressed uniaxially twice to pressures of 50 or 200 kPa with a 2 kN load cell at a rate of 100 kPa minute^−1^ on a DN10 test frame (Davenport-Nene, Wigston, UK) (Gregory *et al.*, 2009) to obtain two batches of 16 cores per field replicate with two different levels of soil bulk density (0.88 ± 0.01 and 0.73 ± 0.01 g dry soil cm^−3^, respectively, *n* = 32). Cores were placed in a tray containing approximately 1–2 cm of deionized water for 2 days and then half were allowed to drain for 1 day to bring the soils to two levels of WFPS (< 80 and > 80%).

As shown by the OA in Table 2, each core received an application of N but not all received C. Potassium nitrate (KNO_3_), dissolved in ionized water, was the N source and it was applied at rates equivalent to 25, 50 or 75 kg N ha^−1^ (45, 90 and 134 mg N kg^−1^ dry soil, respectively). For the treatments receiving C, glucose or cellulose was applied at a rate equivalent to 125 or 250 kg C ha^−1^ (224 and 448 mg C kg^−1^ dry soil, respectively) when the N rate was 25 kg N ha^−1^, 250 and 500 kg C ha^−1^ (equivalent to 448 and 896 mg C kg^−1^ dry soil, respectively) when the N rate was 50 kg N ha^−1^, and 375 and 750 kg C ha^−1^ (equivalent to 672 and 1344 mg C kg^−1^ dry soil, respectively) when the N rate was 75 kg N ha^−1^. There were also some cores that received N but not C. This provided amendments with C:N (g : g) ratios of 0, 5 and 10. The total quantity of water added with C and N amendments was 5 ml per core. The final percentage of WFPS of cores is shown in Table 3.

**Table 3:** Final WFPS (%) and gravimetric moisture content (%) in soils after treatment application (mean ± SD, *n* = 4)

	WFPS / %			
	200 kPa		50 kPa	
Field replicate	> 80%	< 80%	> 80%	< 80%
1	101 ± 2	75 ± 2	92 ± 2	56 ± 1
2	98 ± 3	78 ± 2	93 ± 3	60 ± 1
	Moisture content / %			
1	42.8 ± 0.7	34.7 ± 0.3	46.8 ± 0.5	34.9 ± 0.2
2	42.0 ± 0.6	35.6 ± 0.2	46.7 ± 0.5	35.9 ± 0.2

In order to measure emissions of GHGs, 32 packed cores (16 per field replicate) were incubated in 1-litre Kilner jars at either 15°C (16 cores) or 25°C (16 cores) in constant temperature cabinets for 16 days to give a total of 32 experimental units. During incubation, the lids of the jars were removed to maintain aerobic conditions in the headspace and the jars plus soil cores were weighed every day and rewetted with deionized water to replace any evaporative losses. Measurements of N_2_O, CH_4_ and CO_2_ emissions were made on days 0, 1, 2, 3, 6, 8, 10, 13 and 16. After sealing each jar gas samples were taken by syringe at 0, 20 and 40 minutes after the lids were replaced: the samples were transferred to pre-evacuated vials (22 ml) for analysis. Gas concentrations were analysed by gas chromatography using a Perkin Elmer Clarus 580 GC with a TurboMatrix 110 headspace auto-sampler fitted with an electron capture detector (for N_2_O analysis) and a flame ionization detector connected to a methanizer (for CO_2_ and CH_4_ analysis) with two identical Elite PLOT Q mega-bore capillary columns (Perkin Elmer, Shelton, Connecticut, USA). After the last gas sampling, the soil cores were analysed for gravimetric soil moisture content, soil mineral N content (N-NH_4_^+^ and N-NO_3_^−^) and water soluble organic carbon (WSOC).

Thirty-two additional cores (with identical treatments to those used for measuring GHG emissions) were destructively sampled, immediately after amendment addition, and analysed for gravimetric soil moisture content, soil mineral N content (N-NH_4_^+^ and N-NO_3_^−^) and WSOC. Ammonium and NO_3_^−^ were analysed after extraction with 2 m KCl (1:2 soil:extractant ratio) by automated colorimetric determination (Kamphake *et al.*, 1967; Searle, 1984) using a segmented flow analyser (Skalar, Skalar SAN^PLUS^, Breda, the Netherlands). Water soluble organic carbon was extracted with Type I water (ASTM classification at 1:5 soil:extractant ratio) and then analysed with a total organic carbon analyser (Skalar Formacs HT CA14 TOC analyser, Breda, the Netherlands)

### Phase III: analysis of the experimental data

Qualitek 4 software for automatic design and analysis of Taguchi experiments (Nutek, Inc., Bloomfield Hills, Michigan, USA) was used to study the following: (i) assessment of the individual influence of each factor on GHG emissions, (ii) analysis of variance (anova), (iii) multiple interactions of the selected factors, (iv) determination of the optimum factor combination (resulting in minimum emissions) and (v) estimation of performance at the optimum condition. The anova was the statistical method used to assess the relationship between each factor and emission of GHGs. The percentage contribution of each significant factor to the total variation was calculated as follows (Ross, 1996):


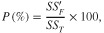
(5)

where *SS_F_*′ is the pure sum of squares due to each factor and *SS_T_* is the total sum of squares and their formulas are:


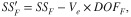
(6)


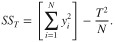
(7)

*DOF_F_* represents the degree of freedom associated with each factor and is calculated by subtracting one from the number of levels of a factor. *SS_F_* is the sum of squares of a specific factor and *V_e_* is the error variance. The general formula for calculating the sum of squares of a specific factor (i.e. factor A) for any number of levels is:


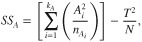
(8)

where *A_i_* is the sum of observations under *A_i_* level, *T* is the sum of all the observations, n_Ai_ is the number of observations under *A_i_* level, *N* the total number of experimental units performed and *k_A_* is the number of levels of factor A. The equation used to calculate *V_e_* is:


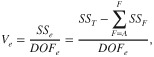
(9)

where *DOF_e_* represents the degree of freedom associated with error and is calculated by subtracting one and *DOF_F_* from the total number of experimental units performed.

Interaction between factors was estimated by calculating a severity index (SI) of the different factors under study (Roy, 2001). For the interaction between factor A and factor C, the generalized formula for the SI is:


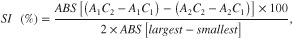
(10)

where *A_1_C_2_* is the average effect for factor A at level 1 and factor C at level 2 and is calculated by averaging results that contain the effects of both A_1_ and C_2_ and similarly for *A_1_C_1_*, *A_2_C_2_* and *A_2_C_1_*. The strength of the interaction can be measured in terms of a numerical quantity that measures the angle between the two lines. The SI is formed such that it is 100% when the lines are perpendicular and 0% when the lines are parallel. The interaction exists when the lines are non-parallel.

Simple linear correlation analyses were performed by using SPSS 15.0 (Chicago, USA) to study the relationships between N_2_O, CH_4_ or CO_2_ fluxes and soil properties (percentage of WPFS and NO_3_^−^, NH_4_^+^ and WSOC concentrations) 1 and 16 days after application of amendment. Total GHGs as CO_2_ equivalents was calculated using the following equation (IPCC, 2006):


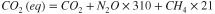
(11)

## Results

### Relative influence of individual factors

The GHG emissions were very much dependent on the selected factors and their ‘levels’ (Table 2). The average effects of the factors at the assigned levels for each GHG cumulative flux and total GHG are shown in Table 4 and Figures S1–S4 in Supporting Information. The difference between averages of values at levels 2 and 1 (L2 minus L1) of each factor is an estimate of the relative influence of the factors on the variability of the GHG's emission process. The larger the difference the stronger is the influence. According to this criterion our results showed that all factors at level two resulted in an increase in N_2_O emissions, although WFPS was the most important factor controlling N_2_O emission at an individual level. Table 4 and Figure S1 also show that N_2_O cumulative fluxes first increased and then decreased when the C:N ratio was largest, regardless of the C source used (glucose and cellulose).

**Table 4:** Main effects of individual factors at assigned levels on cumulative GHG emissions after 16 days

Factors	Level 1	Level 2	Level 3	L2–L1
N_2_O cumulative fluxes / µg N kg^−1^ dry soil				
Nitrate	4630	7108	27 621	2479
Glucose	11 470	14 595	6452	3125
Cellulose	8451	19 084	8003	10 633
Temperature	5817	16 177	—	10 361
WFPS	254	21 740	—	21 485
Soil compaction	6784	15 210	—	8426
CH_4_ cumulative fluxes / µg C kg^−1^ dry soil				
Nitrate	101	186	178	85
Glucose	92	111	270	19
Cellulose	127	153	159	26
Temperature	105	177	—	72
WFPS	139	142	—	3
Soil compaction	171	112	—	−59
CO_2_ cumulative fluxes / mg C kg^−1^ dry soil				
Nitrate	453	487	671	35
Glucose	398	547	721	150
Cellulose	514	475	561	−39
Temperature	391	640	—	249
WFPS	526	503	—	−24
Soil compaction	557	475	—	−82
Total GHG as CO_2_ equivalents / mg C kg^−1^ dry soil				
Nitrate	1889	2695	9237	806
Glucose	3954	5074	2727	1120
Cellulose	3136	6394	3045	3258
Temperature	2196	5659	—	3462
WFPS	610	7245	—	6635
Soil compaction	2663	5192	—	2529

Nitrate addition, glucose at the largest addition and soil temperature were the most important individual factors increasing CH_4_ emission, although for this gas, soil compaction resulted in a decrease of total fluxes (Table 4 and Figure S2). In the case of soil respiration (CO_2_ cumulative fluxes), fluxes were enhanced by, in order, soil temperature, glucose addition and nitrate addition, but decreased by cellulose, WFPS and soil compaction when level two of the factors was applied to the soil cores. However, those fluxes also showed an important increase when the largest dose of glucose, nitrate and cellulose was added (Figure S3).

When considering the total GHGs as CO_2_ equivalents (total of N_2_O, CO_2_ and CH_4_), all factors at level two increased the flux, whilst the largest C:N ratio decreased them (Figure S4). In general, when fluxes of N_2_O were small, CO_2_ fluxes contributed more to the total CO_2_ equivalent flux, whilst large fluxes of CO_2_ equivalents were dominated by the N_2_O contribution.

### Analysis of variance (anova)

**Figure 1:**
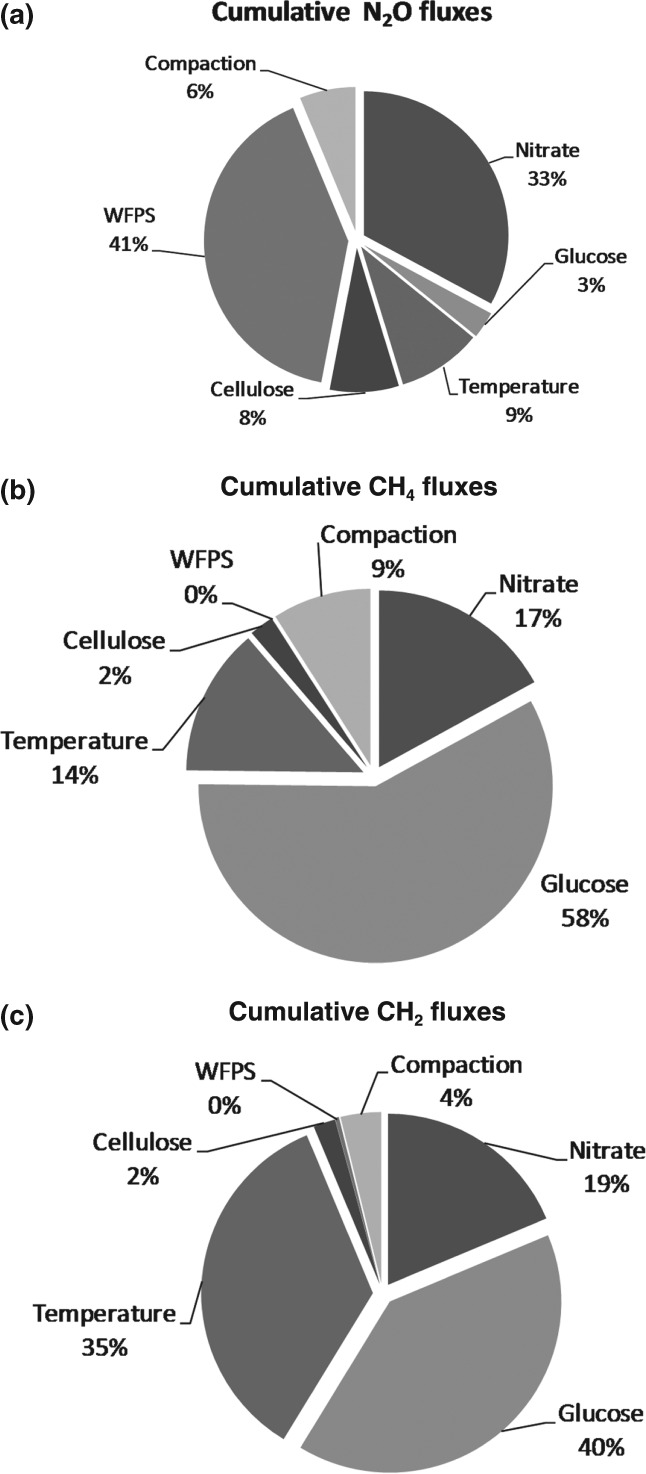
Percentage contribution of each individual factor to the cumulative fluxes of N_2_O (a), CH_4_ (b) and CO_2_ (c).

**Figure 2:**
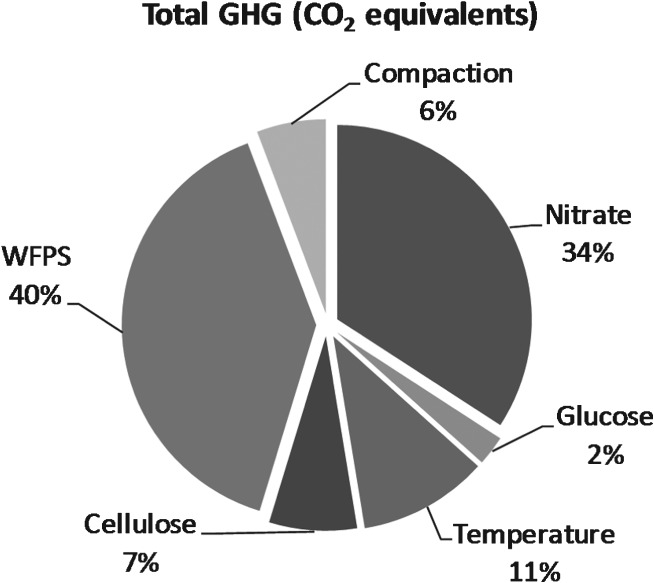
Percentage contribution of each individual factor to total cumulative GHG fluxes expressed as CO_2_ equivalents.

Analysis of variance was used to analyse the results of the experiment and to determine the percentage of contribution of each individual factor to the variation in emissions. All the factors considered had statistically significant effects at the 95% confidence limit. The anova table for N_2_O (Table 5) is included as an example and the percentage contribution of each factor on N_2_O, CH_4_ and CO_2_ total fluxes, and the sum of the three gases totals in CO_2_ equivalents are shown in Figures 1 and 2, respectively.

**Table 5:** Example table for analysis of variance (anova) for cumulative N_2_O fluxes

Factors	DOF	Sum of squares (SS)	Variance (V)	F-ratio	Pure sum (SS′)	Per cent P / %
Nitrate	2	1 490 141 514.56	745 070 757.28	4 470 480 055 223	1 490 141 514.56	32.9
Glucose	2	136 217 580.06	68 108 790.03	40 861 219 1791	136 217 580.06	3.0
Cellulose	2	349 282 303.06	174 641 151.53	1 047 905 725 828	349 282 303.06	7.7
Temperature	1	429 370 201.56	429 370 201.56	2 576 196 080 706	429 370 201.56	9.5
WFPS	1	1 846 528 326.56	1 846 528 326.56	11 078 965 696 109	1 846 528 326.56	40.7
Soil Compaction	1	283 930 925.06	283 930 925.06	1 703 633 704 778	283 930 925.06	6.3
Other/error	6	−0.001	−0.001	—	—	0.002
Total	15	4 535 470 850.86	—	—	—	—

### Optimum levels of the factors for achieving minimum GHG emissions

From the average effects of the factors (Table 4), data were explored to determine the conditions that resulted in the minimum GHG emissions (Table 6). The expected results at optimum conditions were also estimated (Table 6). For N_2_O, the smallest emissions were as indicated in Table 6 and Figure 1(a); WFPS at < 80% and the smallest NO_3_^−^ addition (25 kg ha^−1^) were the dominant factors and levels.

**Table 6:** Optimum conditions for minimum GHG emissions and the contribution of different factors at optimized conditions. The contribution value is the amount of improvement obtained when factor setting is at the adequate level for minimum emissions. This value is calculated in relation to the grand average shown in Table 1. The expected result at optimum conditions is an estimate of performance at optimum conditions. This value is calculated by adding the contribution from all factors to the grand average shown in Table 1

Factors	Level description	Level	Contribution
Cumulative N_2_O fluxes / µg N kg^−1^ dry soil			
Nitrate	Low (25 kg N ha^−1^)	1	−6367
Glucose	High (C:N = 10)	3	−4545
Cellulose	High (C:N = 10)	3	−2994
Temperature	Low (15°C)	1	−5180
WFPS	< 80%	1	−10 743
Soil compaction	Low (50 kPa)	1	−4212
*Expected result at optimum conditions*		−*23 045*	
Cumulative CH_4_ fluxes / µg C kg^−1^ dry soil			
Nitrate	Low (25 kg N ha^−1^)	1	−40.4
Glucose	Low (C:N = 0)	1	−49.4
Cellulose	Low (C:N = 0)	1	−14.5
Temperature	Low (15°C)	1	−36.1
WFPS	< 80%	1	−1.4
Soil compaction	High (200 kPa)	2	−29.5
*Expected result at optimum conditions*		−*30.0*	
Cumulative CO_2_ fluxes / mg C kg^−1^ dry soil			
Nitrate	Low (25 kg N ha^−1^)	1	−63.3
Glucose	Low (C:N = 0)	1	−118.1
Cellulose	Medium (C:N = 5)	2	−40.9
Temperature	Low (15°C)	1	−124.4
WFPS	> 80%	2	−13.1
Soil compaction	High (200 kPa)	2	−41.1
*Expected result at optimum conditions*		*114.7*	
Total GHG as CO_2_ equivalents / mg C kg^−1^ dry soil			
Nitrate	Low (25 kg N ha^−1^)	1	−2038
Glucose	High (C:N = 10)	3	−1201
Cellulose	High (C:N = 10)	3	−882
Temperature	Low (15°C)	1	−1731
WFPS	< 80%	1	−3317
Soil compaction	Low (50 kPa)	1	−1264
*Expected result at optimum conditions*		−*6507*	

In the case of minimum CH_4_ emissions (Table 6 and Figure 1b), the small glucose addition had the major effect (58%), whilst WFPS at < 80% had only a small effect (0.02%) at their individual levels. For minimum CO_2_ emissions (Table 6 and Figure 1c), the most influential factors were glucose and soil temperature, accounting together for 75% of the overall variance of the experimental data. For the minimum CO_2_ equivalents (Table 6 and Figure 2), the most influential factors at their individual levels were the percentage of WFPS and NO_3_^−^ concentration: this was similar to the effects on minimum N_2_O emissions (explaining 74% of the overall variance).

### Interaction of factors

When the interaction of different factors was calculated for each gas (Table 7), it was interesting to note that the less important individual factors, such as glucose and soil compaction (at their individual levels) for N_2_O and CO_2_ equivalent had the largest severity index percentage (SI) when combined. Hence, these factors had little impact on GHG emissions on their own, but had greater impact when acting jointly. The larger the SI value the larger the impact on the particular process as combined factors. For N_2_O, glucose with cellulose (both at level 2, C:N = 5) and glucose with soil compaction (both at level 1, C:N = 0 and 50 kPa, respectively) gave SI values of 68.8 and 68.6%, respectively. In the case of CH_4_ emissions (Table 7), temperature (at level 1, 15°C) when interacting with soil compaction (at 50 kPa) or cellulose (at C:N = 5) resulted in the largest SI (57.7 and 53.0%, respectively). For CO_2_ emissions (Table 7), soil compaction played a greater role when interacting with other factors. Thus the largest SI was found for soil compaction (at 50 kPa) in combination with percentage of WFPS at > 80% (67.8%), soil temperature at 15°C (48.7%) and glucose at C:N = 0 (48.0%). When the presence of interactions among factors was studied for total GHGs as CO_2_ equivalents, the interacting factor pairs found (in order of SI) were the same as those found for cumulative N_2_O fluxes.

**Table 7:** Estimated interaction of severity index (SI) between two factors for cumulative N_2_O, CH_4_ and CO_2_ fluxes after 16 days and total GHG as CO_2_ equivalents

Interacting factor pairs	Severity index (SI) / %	Factor levels for the two factors (respectively) desirable for the optimum condition for minimum emissions
Cumulative N_2_O fluxes		
Glucose × cellulose	68.8	L2 × L2
Glucose × soil compaction	68.6	L1 × L1
Cellulose × soil compaction	55.9	L2 × L1
Temperature × cellulose	41.9	L1 × L2
Cumulative CH_4_ fluxes		
Temperature × soil compaction	57.7	L1 × L1
Nitrate × WFPS	56.9	L1 × L1
Cellulose × WFPS	55.6	L2 × L2
Temperature × cellulose	53.0	L1 × L2
Cumulative CO_2_ fluxes		
WFPS × soil compaction	67.8	L2 × L2
Temperature × soil compaction	48.7	L1 × L1
Glucose × soil compaction	48.0	L1 × L1
Nitrate × WFPS	46.5	L2 × L2
Total GHG as CO_2_ equivalents		
Glucose × soil compaction	71.1	L1 × L1
Glucose × cellulose	70.4	L2 × L2
Cellulose × soil compaction	56.0	L2 × L1
Temperature × cellulose	41.5	L1 × L2

### Soil properties

Although an identical amount of water was added to each soil core for the same level of soil moisture, WFPS immediately after water application was around 98 and 92%, respectively, in compacted and less compacted soils for the large moisture content and about 78% (compacted) and 58% (less compacted) for soil cores at the smaller moisture content (Table 8).

**Table 8:** Soil WFPS (%), NO_3_^−^ (mg N-NO_3_^−^ kg^−1^ dry soil), NH_4_^+^ (mg N-NH_4_^+^ kg^−1^ dry soil) and water soluble organic carbon (WSOC, mg C kg^−1^ dry soil) contents in the experimental units 1 day after amendment applications (1DAA) and at the end of the experiment (16 DAA) (mean ± SD, *n* = 2)

	1 DAA WFPS	16 DAA WFPS	1 DAA NO_3_^−^	16 DAA NO_3_^−^	1 DAA NH_4_^+^	16 DAA NH_4_^+^	1 DAA WSOC	16 DAA WSOC
Experimental unit	/ %		/ mg N kg^−1^ dry soil		/ mg N kg^−1^ dry soil		/ mg C kg^−1^dry soil	
1	58.1 ± 2.8	59.5 ± 3.2	36.9 ± 5.4	38.2 ± 1.1	2.6 ± 0.9	8.2 ± 1.5	40.2 ± 7.3	40.3 ± 9.1
2	76.7 ± 2.6	72.6 ± 1.5	43.3 ± 2.5	37.0 ± 1.2	1.9 ± 0.4	5.7 ± 1.6	34.0 ± 0.8	36.1 ± 3.0
3	91.9 ± 0.5	90.5 ± 1.7	1.1 ± 0.8	0.6 ± 0.8	8.1 ± 1.8	28.9 ± 11.6	57.0 ± 13.2	103 ± 12
4	98.3 ± 4.5	99.9 ± 2.0	44.1 ± 0.7	2.9 ± 2.0	8.7 ± 4.5	44.6 ± 10.6	47.6 ± 8.7	70.0 ± 8.0
5	92.0 ± 0.4	90.8 ± 1.2	77.4 ± 6.5	24.3 ± 5.1	8.6 ± 2.2	18.0 ± 0.5	41.7 ± 13.8	57.4 ± 7.4
6	99.1 ± 3.1	101 ± 3	79.7 ± 0.4	1.1 ± 0.4	4.1 ± 2.7	21.6 ± 5.4	42.4 ± 0.2	74.9 ± 11.4
7	58.4 ± 4.7	59.2 ± 1.6	53.6 ± 13.0	58.8 ± 2.2	1.5 ± 0.2	16.6 ± 6.9	36.6 ± 0.6	36.1 ± 2.7
8	78.5 ± 4.1	74.7 ± 3.2	86.3 ± 8.4	78.6 ± 1.3	4.4 ± 1.6	30.0 ± 7.1	38.4 ± 17.5	37.4 ± 9.6
9	98.9 ± 3.0	101 ± 2	109 ± 11	0.8 ± 0.6	8.4 ± 3.3	41.4 ± 8.4	31.7 ± 6.1	86.2 ± 4.1
10	92.9 ± 0.6	96.1 ± 3.8	90.2 ± 11.2	0.1 ± 0.2	8.5 ± 0.1	16.8 ± 3.6	44.4 ± 5.8	136 ± 24
11	78.1 ± 3.3	76.4 ± 0.1	132 ± 0	105 ± 2	1.2 ± 0.1	6.3 ± 2.9	21.9 ± 2.3	27.8 ± 2.8
12	58.1 ± 3.0	58.6 ± 2.6	116 ± 20	122 ± 5	4.5 ± 1.6	13.3 ± 2.4	27.7 ± 10.0	28.9 ± 11.3
13	79.1 ± 0.3	75.1 ± 2.8	43.8 ± 4.3	37.6 ± 2.5	3.7 ± 0.7	14.9 ± 2.3	41.8 ± 3.0	37.7 ± 2.9
14	59.6 ± 2.8	58.3 ± 1.2	38.8 ± 5.5	37.3 ± 1.6	1.8 ± 0.7	13.8 ± 3.5	26.9 ± 12.1	34.3 ± 2.6
15	99.6 ± 3.4	102 ± 5	39.2 ± 1.0	2.7 ± 1.8	3.3 ± 1.9	16.1 ± 6.8	44.7 ± 10.2	61.8 ± 10.5
16	92.9 ± 1.7	94.4 ± 1.7	36.5 ± 1.1	0.8 ± 1.1	7.6 ± 1.5	16.6 ± 0.6	38.1 ± 4.4	81.3 ± 0.4

The initial mineral N content of the soil before any treatment application was 6.0 mg N-NO_3_^−^ kg^−1^ dry soil and 1.5 mg N-NH_4_^+^ kg^−1^ dry soil. The application of amendments resulted in an increase in soil mineral N (Table 8), and for NO_3_^−^ content 1 day after application of amendment (1 DAA) this was dependent on the amount of KNO_3_ added.

The initial WSOC in soil before any treatment application was 46 mg C kg^−1^ dry soil. The addition of glucose or cellulose did not enhance WSOC at 1 DAA, or at 16 DAA except in soil cores at the greater WFPS (Table 8).

The cumulative emission of N_2_O was linearly correlated with WFPS at 1 DAA and 16 DAA (respectively, *R*^2^ = 0.590 and 0.596, *P* < 0.05) and with WSOC at 16 DAA (*R*^2^ = 0.618, *P* < 0.05). Significant correlations among cumulative CO_2_ and CH_4_ fluxes and soil properties were not found.

## Discussion

The Taguchi methodology provides an excellent opportunity to study important interactions between controlling variables of GHG production in and emission from soil. Through this increased understanding of soil processes it may allow researchers to develop and test strategies to mitigate GHG emissions in agro-ecosystems. The advantage of the Taguchi methodology is that a large number of factors can be studied in the same laboratory experiment with a small number of combinations, providing enough information about their mutual interactions. In a conventional statistical, full factorial design using six different factors with two and three different rates or levels for three factors each, it is necessary to have 2^3^ × 3^3^ (216) different combinations. With the DOE design, the number of combinations required to obtain equivalent information as described by the OA (L_16_) was 16 as in this experiment. Exploration of the data allows researchers to determine the optimized conditions (within the given levels used in the experiment) that generate the minimal flux, and hence provide essential information on which to base mitigation practices in an effective way. These recommendations would then need to be confirmed with field experiments comparing traditional management with the best possible options. The results from our experiment were consistent with those found in the published literature for each GHG where information was available for a limited number of factors and/or levels, in other words for one or two factors-levels combinations.

### Nitrous oxide

The factors controlling N_2_O fluxes were consistent with effects on denitrification as the dominant process (instead of nitrification). The addition of NO_3_^−^ as an exogenous source of N, the addition of labile C sources, used by heterotrophic microorganisms, and a WFPS > 60% (except for three of the present treatments) favour denitrification (Davidson, 1991). Our results indicated that the percentage of WFPS was the main factor involved (explaining 41% of the variance, Figure 1a) and must be maintained below 80% (Table 6) in order to reduce N_2_O emission: this is in line with other findings (Meixner & Yang, 2006; Schaufler *et al.*, 2010). Although in rain-fed crops, moisture content cannot be controlled by farmers, other practices such as fertilizer input and cultivation could be synchronized, selecting the best moisture content to reduce N_2_O emission. More possibilities exist in irrigated crops, in which irrigation management could be used to control soil moisture content.

The amount of NO_3_^−^ applied as fertilizer was also an essential factor in controlling N_2_O emission and explained 33% of the variance (Figure 1a). The increase in N emission was not linearly related to the N addition (Table 4, Figure S1). Thus, the difference in fluxes between L1 and L2 (representing an N addition of 25 kg N ha^−1^) was 2.48 mg N kg^−1^, whereas that between L2 and L3 (also an increase in the N addition of 25 kg N ha^−1^) was 20.5 mg N kg^−1^ (Table 4). A non-linear relationship has been observed previously for this soil type (Cárdenas *et al.,* 2010), and these results are consistent with our knowledge of enzyme functioning. At large NO_3_^−^ concentrations inhibition of N_2_O reductase activity occurs (Cárdenas *et al.*, 2003; Bergstermann *et al.*, 2011) because of the competitive effect of NO_3_^−^ and N_2_O as electron acceptors during denitrification.

The addition of an exogenous C source, such as glucose (soluble and easily degradable) or cellulose (non-soluble and gradually degradable), together with NO_3_^−^, produced a contradictory effect on emission. The application of a small amount of C relative to N (C:N = 5) enhanced emission when compared with no addition (level 1, C:N = 0), but mitigated fluxes at a larger application (C:N = 10, Table 4), regardless of the quality of added C (glucose or cellulose). The availability of C not only supports the activity of denitrifiers *per se*, but also has the indirect effect of causing micro-site anaerobiosis, because of increased respiratory demand for O_2_. Increased availability of labile C will favour complete denitrification to N_2_, as also observed by Sánchez-Martín *et al.* (2008) when N was applied with glucose to soil.

The combination of glucose × cellulose (at level 2 for both factors) gave the largest SI values (68.8% in this case; C:N = 10, Table 7). An explanation for this could be that glucose could prime the use of cellulose (insoluble) by microorganisms. The combination of both sources of C probably best met the need of C by heterotrophic microorganisms in the experimental period and maintained a large demand for electrons, which contributed to N_2_O consumption and N_2_ production. Therefore, strategies based on the application of a labile C source in combination with N, such as occurs with organic fertilizers or crop residues mixed with N fertilizers, must be taken into account and, as occurred in this soil, this could have the potential to reduce N_2_O emission.

Soil compaction also affected fluxes, increasing from 6784 µg kg^−1^ (less compaction) to 15 210 µg kg^−1^ (more compaction) (see Table 4), although the relative importance of this factor in relation to others was not great (6.3%). Level 1 compaction represented a static load exerted, for example, by a sheep and level 2 that of heavy machinery (Gregory *et al.*, 2007) or cow hooves (Scholefield *et al.*, 1985). Increased N_2_O emissions in compacted soils have been attributed to reduced gas diffusivity and air-filled porosity (Skiba *et al.,* 2002). Extrapolating our results to soil tillage, it could be expected that soil tillage reduces soil compaction (as with level 1) and helps to reduce emissions (Menéndez *et al.*, 2008). However, optimum conditions for this soil could occur when residues with substantial amounts of labile C are incorporated by ploughing, because the interaction between glucose and soil compaction (SI = 68.6%) and between cellulose and soil compaction (SI = 55.9%) resulted in less emission (Table 7).

Temperature also increased N_2_O fluxes. An increase of 10°C enhanced the average cumulative N_2_O fluxes by 2.8 times (Table 4). Under denitrifying conditions, a Q_10_ (the factor by which the denitrification rate differs for a temperature interval of 10°C) value close to or greater than three has been estimated frequently (Maag & Vinther, 1999).

### Methane and carbon dioxide

Glucose was the main factor to influence CH_4_ and CO_2_ fluxes, increasing emissions in line with the rate of C applied. Glucose is a labile source that could be easily used by methanogenic bacteria to produce CH_4_ and by other microorganisms producing CO_2_. The other source of C applied in this soil, cellulose, had a small effect on emission of CH_4_ and CO_2_ fluxes, probably because of its slow rate of degradation in soil.

Soil temperature is one of the most important regulators of ecosystem respiration (Silvola *et al.*, 1996). In our experiment an increase of 10°C enhanced soil respiration and CH_4_ emission by 1.6 and 1.7 times, respectively (Table 4, Figures S3 and S2). This variation is consistent with Q_10_ values found for biological processes, which range from 1.3 to 3.3 in reviews of soil respiration (Tjoelker *et al.*, 2001).

Minimization of CH_4_ requires soil conditions that promote methanotrophic rather than methanogenic activity. As expected, this was favoured by low temperature, a smaller percentage of WFPS (increasing soil aeration) and no application of C to soil (Tables 4, 6). Nitrate suppresses CH_4_ production in anoxic soil (Roy & Conrad, 1999), because methanogenesis is inhibited by denitrification intermediates (NO_2_^−^, N_2_O and NO). However, for methanotrophic bacteria, the addition of large amounts of NO_3_^−^ also inhibits CH_4_ oxidation (Rigler & Zechmeister-Boltenstern, 1999), probably because of an increase in osmotic potential (Hütsch *et al.*, 1996). This may have occurred in our experiment when the rate of added NO_3_^−^ was increased. Our results confirmed that the optimum conditions to mitigate CH_4_ fluxes from this soil were when NO_3_^−^ was applied at the smallest rate (25 kg N ha^−1^). Soil compaction and soil moisture only had small effects on CH_4_ emission, which was contrary to our expectation, because more anaerobic sites often promote methanogenesis and reduce oxidation.

The application of mineral N generally increases CO_2_ fluxes (Iqbal *et al.*, 2009). In our experiment, a non-linear increase was found (L1–L2 = 35 mg C kg^−1^ whereas L1–L3 = 184 mg C kg^−1^, Table 4 and Figure S3). This increase after N addition was probably produced by a priming effect of fertilizer, which often promotes a rapid mineralization of soil organic matter (Kuzyakov, 2002).

### Total GHG

Total GHG, expressed as CO_2_ equivalent, was very dependent on N_2_O emission, except when WFPS was reduced, when CO_2_ had a larger influence. Therefore, its reduction must be related to mitigation of N_2_O emission. Other factors not included in this study could also affect emissions, for example the use of NH_4_^+^ or urea-based fertilizers, other C sources with different availability for microorganisms, a wider variation of WFPS and the presence of plants.

From a methodological point of view, the use of repacked soil cores for this type of study is recommended, because it reduces the inherent variability of soil factors and their effect on GHG emissions, thus allowing treatment effects to dominate gaseous fluxes. This implies that the Taguchi methodology is most suitable for laboratory-based experiments, or where uniform soil properties would be expected in the field, such as arable-tilled soils or homogenous peat soils, but not those from grazed grassland.

## Conclusions

The Taguchi methodology offers new possibilities to assess complex interactions between factors known to control soil processes and, in the present case, can be used to help establish strategies to mitigate GHG emissions from soil. A large number of factors can be evaluated in order to quantify their relative effect on emissions, but using a reduced number of combinations in comparison with those used in an equivalent statistical factor design. In our experiment, five of the most commonly defined factors affecting emissions were selected and results were consistent with those found in published studies where, in general, conventional experimental procedures had been used that adjusted one variable at a time.

Although other possible factors and levels could have been studied, our results indicated that total GHG emissions from agricultural soils, expressed as CO_2_ equivalents, were dependent on N_2_O emissions, where the percentage of WFPS and addition of NO_3_^−^ were the main influencing factors.

Further studies using other possible factors and rates not included in our experiment should be investigated. It would also be of value to validate under field conditions the optimized conditions that generate minimal and even maximal fluxes obtained with the laboratory-based Taguchi methodology.

## Supporting Information

The following supporting information is available in the online version of this article:

**Figure S1.** Plot of factor average effects of N_2_O cumulative fluxes. The dashed line represents the grand average, the average of all the observations. A line with a small angle or which is horizontal has less influence on the variability of the emission process. The red circles represent the optimum conditions based on the quality characteristic ‘smaller is better’. Error bars represent the standard error of the mean.

**Figure S2.** Plot of factor average effects of CH_4_ cumulative fluxes. The dashed line represents the grand average, the average of all the observations. A line with a small angle or which is horizontal has less influence on the variability of the emission process. The red circles represent the optimum conditions based on the quality characteristic ‘smaller is better’. Error bars represent the standard error of the mean.

**Figure S3.** Plot of factor average effects of CO_2_ cumulative fluxes. The dashed line represents the grand average, the average of all the observations. A line with a small angle or which is horizontal has less influence on the variability of the emission process. The red circles represent the optimum conditions based on the quality characteristic ‘smaller is better’. Error bars represent the standard error of the mean.

**Figure S4.** Plot of factor average effects of total GHG (as CO_2_ equivalents). The dashed line represents the grand average, the average of all the observations. A line with a small angle or which is horizontal has less influence on the variability of the emission process. The red circles represent the optimum conditions based on the quality characteristic ‘smaller is better’. Error bars represent the standard error of the mean.

**TableS1.** Amounts of nitrate (kg N ha^−1^), glucose and celullose (kg C ha^−1^) added at each C:N ratio.

**TableS2.** Physico-chemical properties of the 10 cm of the topsoil layer measured by conventional methods.
