# Supplementary material for: Ranking factors affecting emissions of GHG from incubated agricultural soils
Source: Eur J Soil Sci. 2014 Jun 18;65(4):573–83. doi: 10.1111/ejss.12143 (PMC4146601; doi:10.1111/ejss.12143)
Supplement: Supplementary file 1 — Figure S1. Plot of factor average effects of N2O cumulative fluxes. The dashed line represents the grand average, the average of all the observations. A line with a small angle or which is horizontal has less influence on the variability of the emission process. The red circles represent the optimum conditions based on the quality characteristic ‘smaller is better’. Error bars represent the standard error of the mean. [file ejss0065-0573-SD1.pdf]

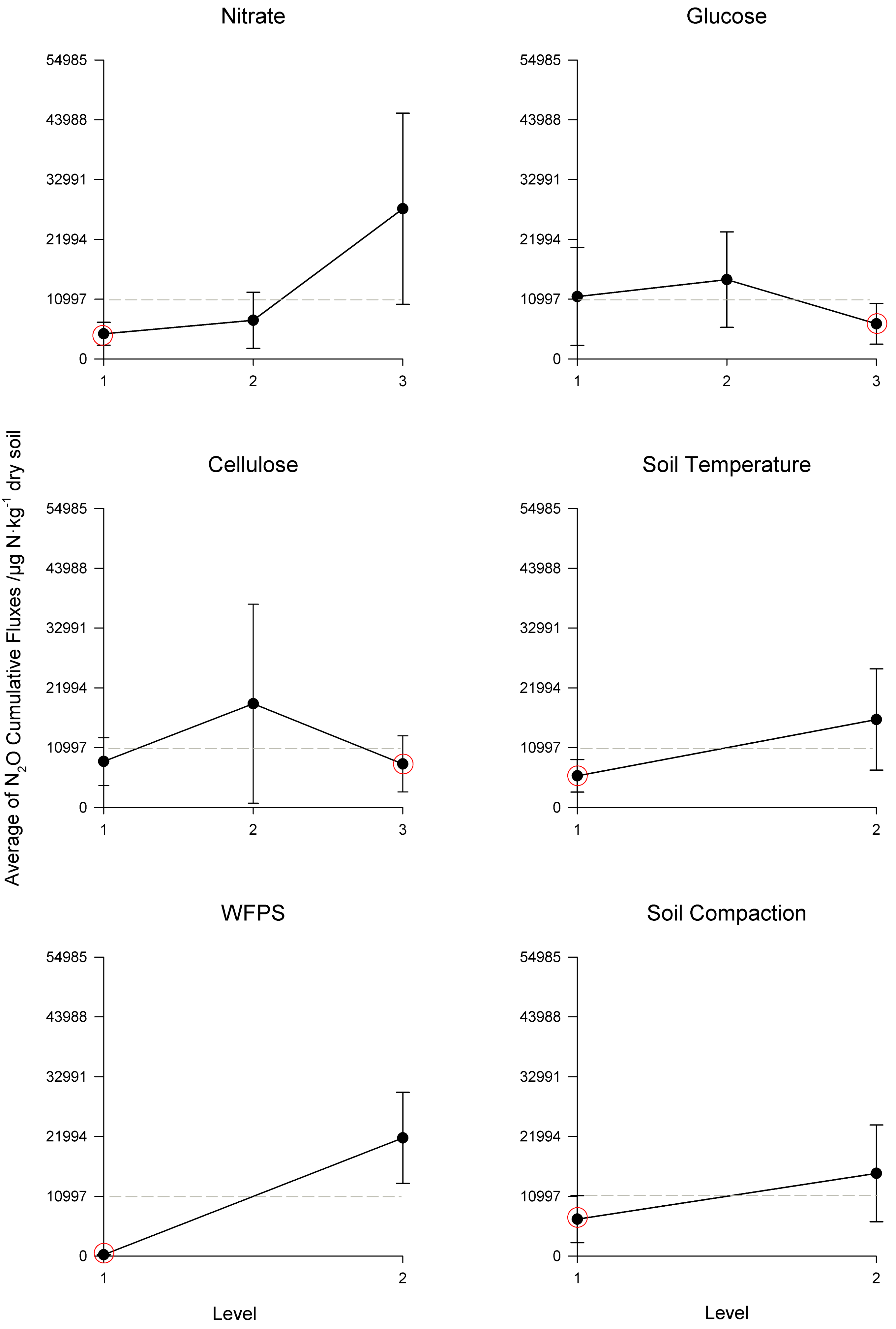

Nitrate
Glucose
Cellulose
Soil Temperature
WFPS
Soil Compaction
54985
43988
32991
21994
10997
0
1
2
3
Average of N_2O Cumulative Fluxes /μg N·kg^{-1} dry soil
Level
