# Supplementary figures and images for: Ranking factors affecting emissions of GHG from incubated agricultural soils
Source: Eur J Soil Sci. 2014 Jun 18;65(4):573–83. doi: 10.1111/ejss.12143 (PMC4146601; doi:10.1111/ejss.12143)

Nitrate

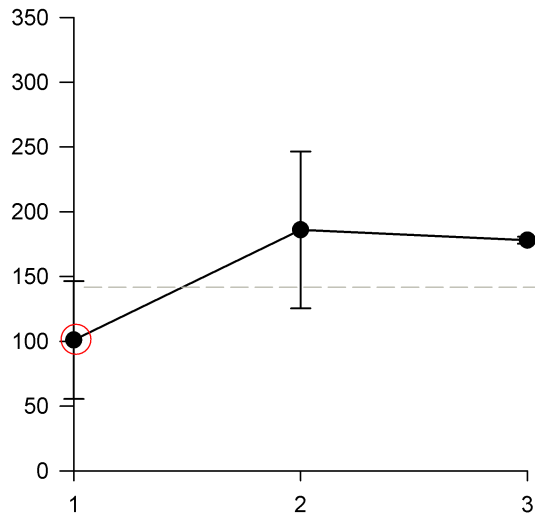

Glucose

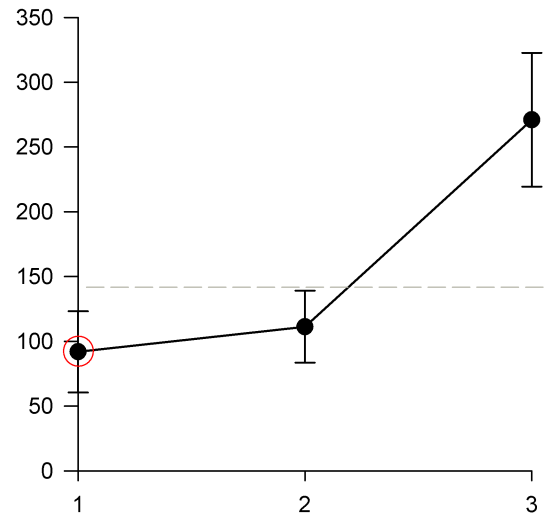

Cellulose

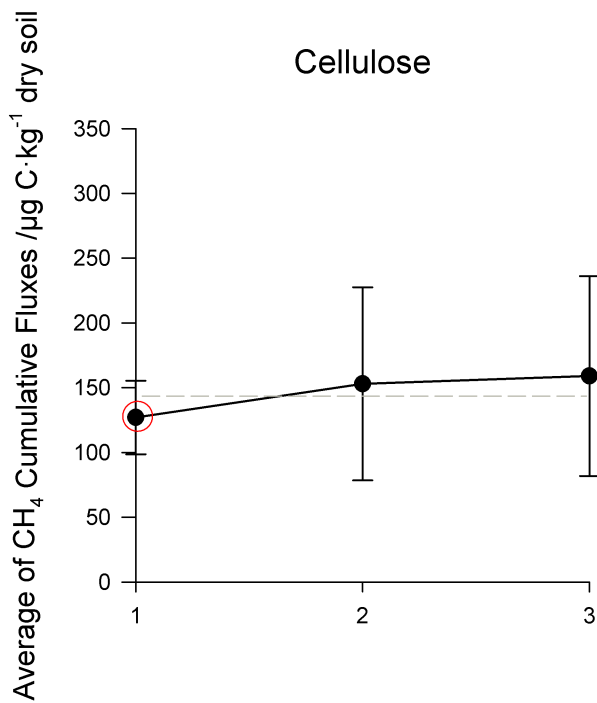

Soil Temperature

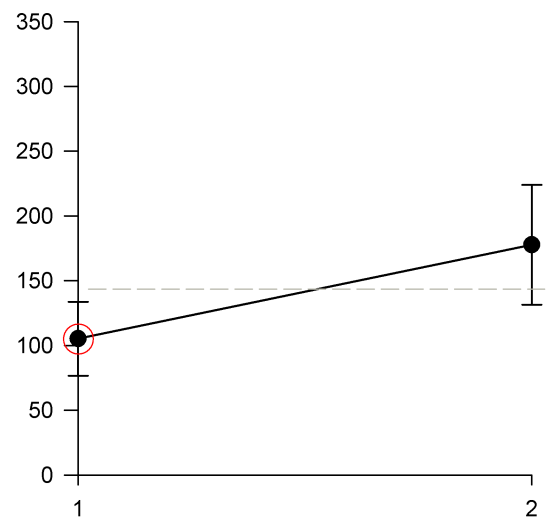

WFPS

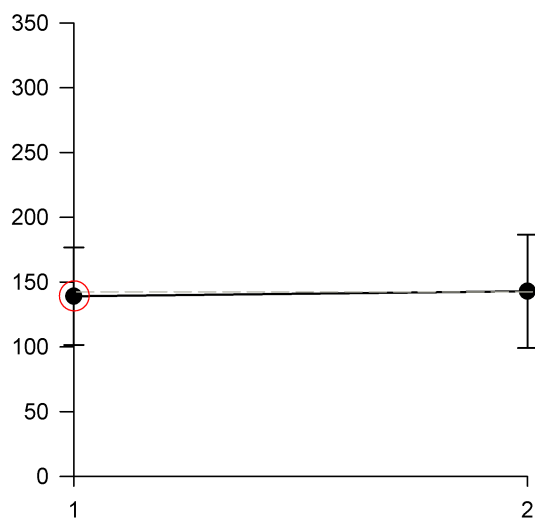

Level

Soil Compaction

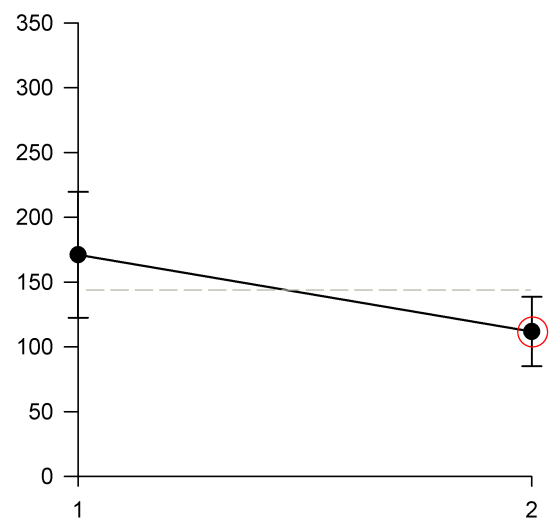

Level

Supplement: Supplementary file 2 — Figure S2. Plot of factor average effects of CH4 cumulative fluxes. The dashed line represents the grand average, the average of all the observations. A line with a small angle or which is horizontal has less influence on the variability of the emission process. The red circles represent the optimum conditions based on the quality characteristic ‘smaller is better’. Error bars represent the standard error of the mean. [file ejss0065-0573-SD2.pdf]

### Nitrate

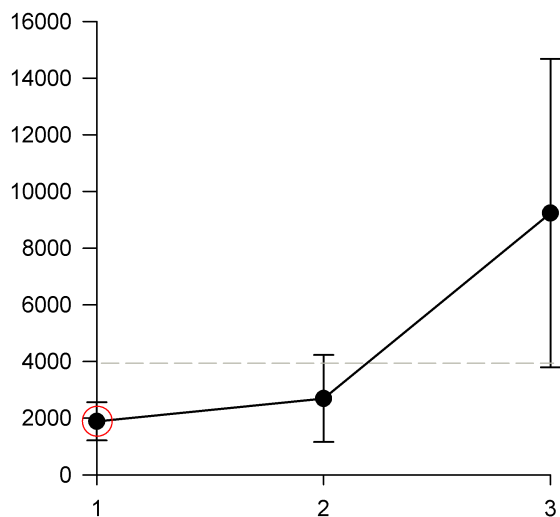

### Glucose

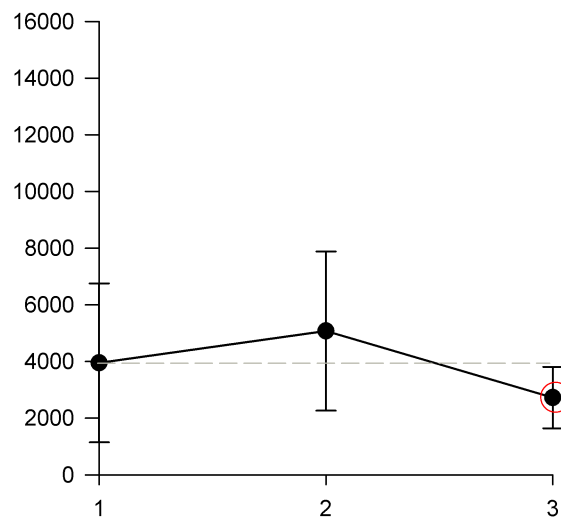

### Cellulose

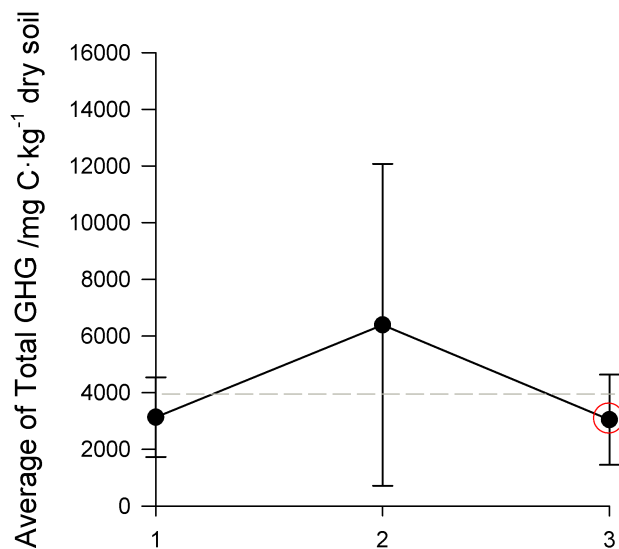

### Soil Temperature

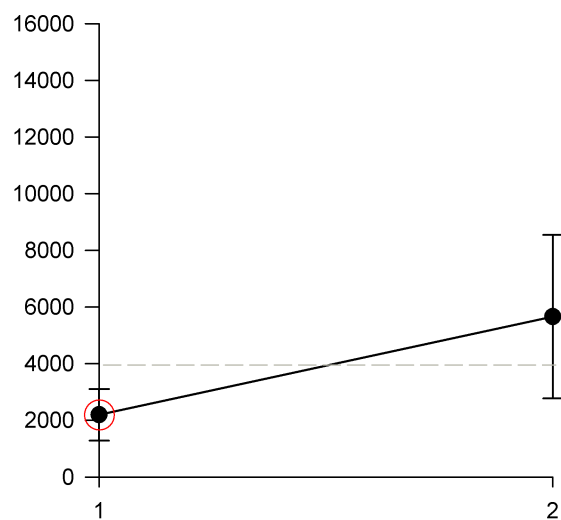

### WFPS

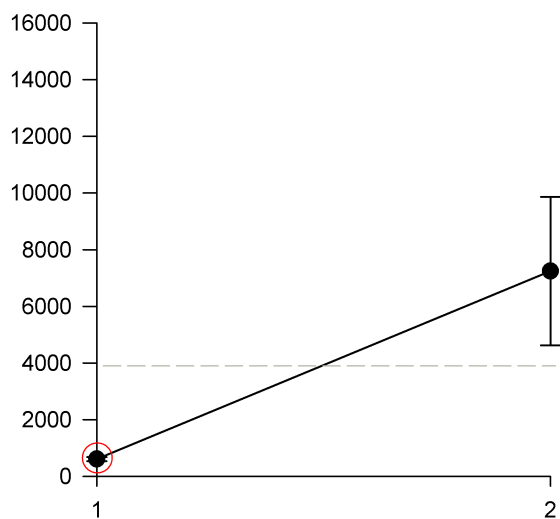

Level

### Soil Compaction

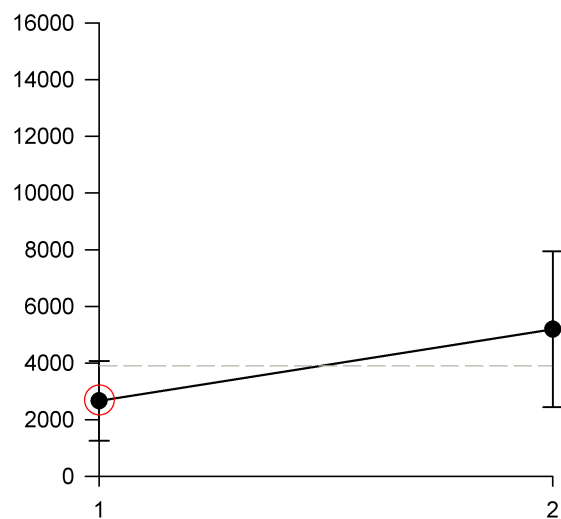

Level

Supplement: Supplementary file 4 — Figure S4. Plot of factor average effects of total GHG (as CO2 equivalents). The dashed line represents the grand average, the average of all the observations. A line with a small angle or which is horizontal has less influence on the variability of the emission process. The red circles represent the optimum conditions based on the quality characteristic ‘smaller is better’. Error bars represent the standard error of the mean. [file ejss0065-0573-SD4.pdf]
