# Supplementary material for: Ranking factors affecting emissions of GHG from incubated agricultural soils
Source: Eur J Soil Sci. 2014 Jun 18;65(4):573–83. doi: 10.1111/ejss.12143 (PMC4146601; doi:10.1111/ejss.12143)
Supplement: Supplementary file 3 — Figure S3. Plot of factor average effects of CO2 cumulative fluxes. The dashed line represents the grand average, the average of all the observations. A line with a small angle or which is horizontal has less influence on the variability of the emission process. The red circles represent the optimum conditions based on the quality characteristic ‘smaller is better’. Error bars represent the standard error of the mean. [file ejss0065-0573-SD3.pdf]

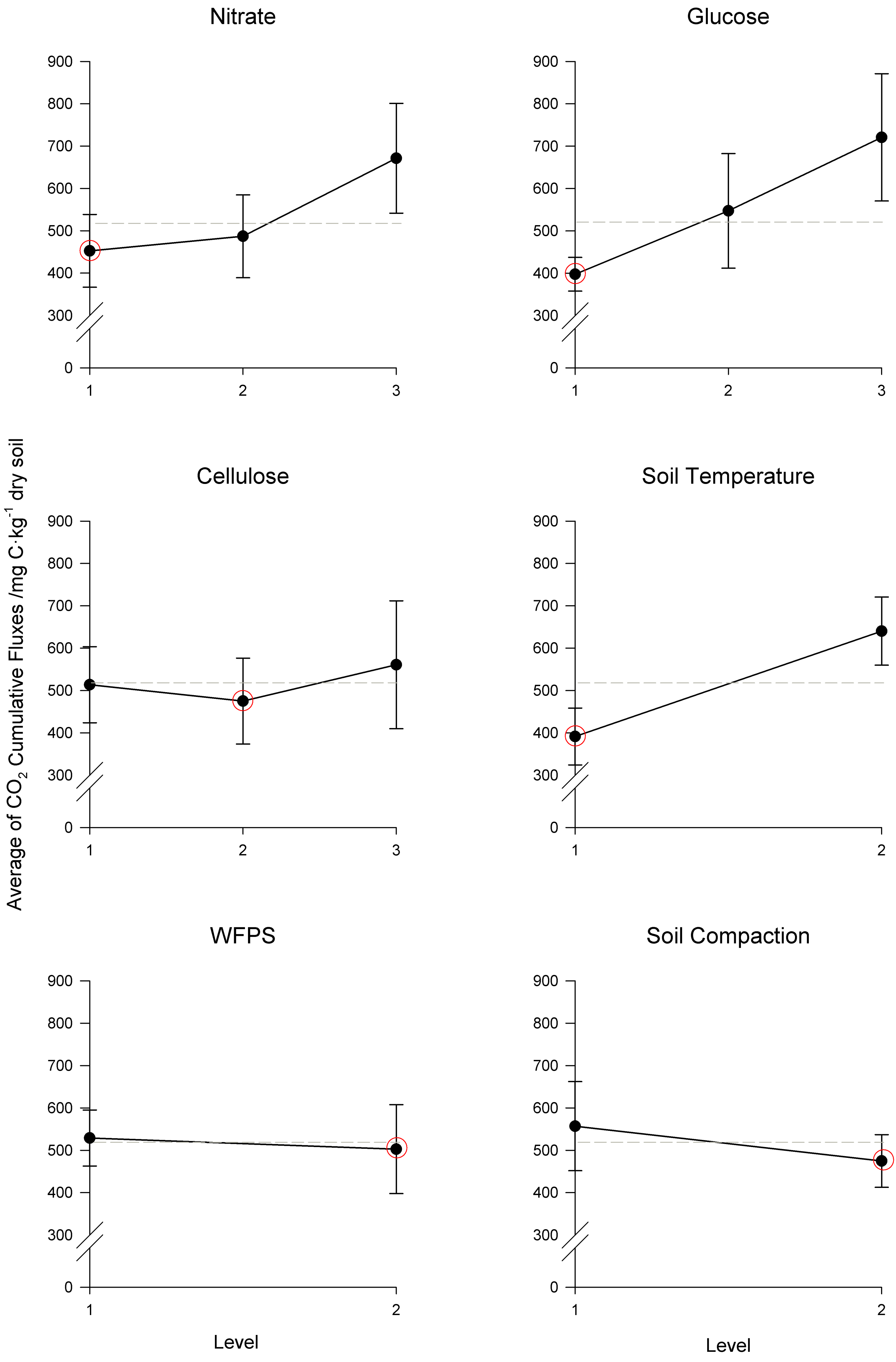

Nitrate
Glucose
Cellulose
Soil Temperature
WFPS
Soil Compaction
Average of CO_2 Cumulative Fluxes /mg C·kg^{-1} dry soil
900
800
700
600
500
400
300
0
1
2
3
Level
