# Supplementary material for: Ranking factors affecting emissions of GHG from incubated agricultural soils
Source: Eur J Soil Sci. 2014 Jun 18;65(4):573–83. doi: 10.1111/ejss.12143 (PMC4146601; doi:10.1111/ejss.12143)
Supplement: Supplementary file 5 — TableS1. Amounts of nitrate (kg N ha−1), glucose and celullose (kg C ha−1) added at each C:N ratio. [file ejss0065-0573-SD5.doc]

**Table S1** Amounts of nitrate (kg N ha^-1^), glucose and celullose (kg C ha^-1^) added at each C:N ratio.

|  | Nitrate /KNO_3_,  kg N ha^-1^ | Glucose /  kg C ha^-1^ | Cellulose /  kg C ha^-1^ |
| --- | --- | --- | --- |
| C/N=0 | 25 | 0 | 0 |
|  | 50 | 0 | 0 |
|  | 75 | 0 | 0 |
| C/N=5 | 25 | 125 | 125 |
|  | 50 | 250 | 250 |
|  | 75 | 375 | 375 |
| C/N=10 | 25 | 250 | 250 |
|  | 50 | 500 | 500 |
|  | 75 | 750 | 750 |
