# Supplementary material for: Ranking factors affecting emissions of GHG from incubated agricultural soils
Source: Eur J Soil Sci. 2014 Jun 18;65(4):573–83. doi: 10.1111/ejss.12143 (PMC4146601; doi:10.1111/ejss.12143)
Supplement: Supplementary file 6 — TableS2. Physico-chemical properties of the 10 cm of the topsoil layer measured by conventional methods. [file ejss0065-0573-SD6.doc]

| **Table S2** Physico-chemical properties of the 10 cm of the top soil layer measured by conventional methods. | | | | | |
| --- | --- | --- | --- | --- | --- |
| Total C_organic_ | pH_H2O_ | Clay | Silt | Fine sand | Coarse sand |
| /% |  | /% | | | |
| 5.3 | 5.7 | 36.6 | 47.7 | 13.9 | 1.8 |
